# Fertilizers and Mixed Crop Cultivation of Chromium Tolerant and Sensitive Plants under Chromium Toxicity

**DOI:** 10.1155/2015/367217

**Published:** 2015-01-29

**Authors:** B. Dheeba, P. Sampathkumar, K. Kannan

**Affiliations:** ^1^Department of Chemistry and Biosciences, Srinivasa Ramanujan Centre, SASTRA University, Kumbakonam 612001, India; ^2^Department of Mathematics, Srinivasa Ramanujan Centre, SASTRA University, Kumbakonam 612001, India

## Abstract

*Zea mays* (maize) and *Vigna radiata* (green gram) are found to be the chromium (Cr) tolerant and sensitive plants, respectively. In the present paper, we investigate the reduction of the toxicity of Cr in the sensitive plants by the mixed crop cultivation in the field using various amendments. Further, the potassium dichromate was used as the source of hexavalent Cr. The results indicated that Cr adversely affects both the growth and yield of plants. The soil properties vary with Cr and different fertilizer amendments and the yield of both plants were affected by Cr. We conclude that metal accumulation of seeds of green gram was higher than corn and the application of single fertilizer either farm yard manure (FYM) or nitrogen, phosphorous, and potassium (NPK) enhances the growth and yield of both the tolerant and sensitive plants in the mixed crop cultivations.

## 1. Introduction

In the present industrialized world, the toxic waste products are released to the environment at each and every moment. Even after recycling and reusing, many of the chemicals find their way to the pollution of environment because of poor planning of waste disposal and treatment and hence the industrial sites and waste disposal sites are contaminated with many pollutants [1, 2]. Among these pollutants heavy metals constitute a major part and they severely affect all the compounds of ecosystem in the soil [3]. Cr is one of the most important such pollutants which enter the ecosystem mainly due to the human activities [4]. In particular, it is observed that Cr(III) and Cr(VI) are the most stable and hexavalent Cr is more toxic [5]. Cr is mainly used due to its properties such as resistance to corrosion, temperature, wear, and decay as well as strength, permanence, hygiene, hardness, and color in the many industries like electroplating, cement, dye, steel, leather tanning, wood, and so forth [4].

Numerous efforts have been taken in recent days for removing heavy metals of soil [6] of which metal-polluted soil can be treated by physical, chemical, or biological methods which are costly [7]. For chemically contaminated soil, vegetation plays progressively more vital ecological and hygienic role [8] and the correct management of green plants in such areas may contribute considerably to restore the natural environment [9]. Phytoremediation is a well matched green tool that can be considered for remediation of impure sites because of its price effectiveness, visual advantages, and long term applicability. Moreover, phytoextraction refers to employing of the plants that take up and concentrate metals from the soil in roots and shoots or foliage [10]. On the other hand, the incidence of oxidative strain possibly will be the result of heavy metal toxicity in sensitive plants and the oxidation of cellular components is caused by the accumulation of molecular oxygen of plants under heavy metal stress [11, 12].

Maize is a resourceful cereal crop that is grown generally all over the world in a choice of agroecological environments. It is capable of phytoextracting metals from polluted soils by transporting them from roots to its other parts [13]. Many researchers have studied heavy metal uptake maize in polluted soils over the time periods, namely, 21 days [14], 30 days [15], 35 days [16], 45 days [17], 60 days, and 90 days [15]. Also, it has broad fibrous roots with huge shoot biomass yield per hectare, withstands unfavorable circumstances, and produces plentiful seeds with ease of cultivation under frequent cropping. The crop is heavy metal tolerant and has elevated metal accumulating ability in the above ground parts with reasonable bioaccumulation factor. These given are capable of constant phytoextraction of metals from impure soils by transferring them from roots to shoots [18].

Green gram, a crucial grain, is grown extensively in tropical countries. It lodges a region of three million hectares, secretarial for 7% of production in total and 14% of the pulses in India [19]. The high metal concentrations affect the growth and nodulation of them and decrease the seed yield. Hence it is considered a metal sensitive plant [20].

Some researchers have conveyed that the use of chemical mobilizing agents, such as ethylene diamine triacetic acid, N-(2-hydroxyethyl)-ethylene diamine triacetic acid, and diethylene-tetramine-penta-acetate acid, can improve the efficiency of phytoextraction [21–23]. In some cases, it may create the possible risk of ground water contamination. The use of fertilizers in phytoremediation has not been explored so far, and little attention is devoted to it in the literature. The use of organic amendments on heavy metal remediation has been influenced by the composition of salt content, organic matter of soil and manure, effects on soil pH, and their effect on microbes, redox potential, and the specific soil and metals concerned [24]. FYM positively controls the crop production [25] and recovers properties of soil and it can be used to decrease heavy metal stress in plants [26].

Thus, an efficient and reasonable scientific resolution is required for farmers to lessen the destructive things of heavy metals in the polluted region. The amendments of FYM and NPK fertilizers by individual and in combination in the Cr polluted and nonpolluted soil were done in the present study in which the mixed crop cultivation of Cr tolerant and sensitive plants using fertilizer amendments was investigated towards the improvement of the growth and yield of heavy metal sensitive plants using tolerant plant and harmless fertilizers.

## 2. Materials and Methods

### 2.1. Study Area and Preparation of Field

In Thirubuvanam near Kumbakonam, Tamil Nadu, India, the well levelled clod and weed free field was set to ensure sufficient plant stand and early vigor. Two ploughings were done for opening up of soil for minimizing the weed and to ensure adequate trapping and protection of moisture. The field consists of 8 small plots, each having an area of 4 m^2^ (2 m × 2 m); four plots are treated with chromium. The potassium dichromate (42 g/plot) was used as a source of Cr (VI) and two replications were maintained. FYM (5 kg/plot) was applied as 12 tons/hectare is the usual amount [27] and NPK was applied at 20 : 10 : 10 (98 g : 49 g : 49 g) levels in field [28]. The fertilizers were mixed with the soil 10 days before planting. Depending upon the wetness of the soil, 2 cm to 3 cm depth is adequate to moisten the soil and 5 cm to 10 cm is optional for dry planting. The depth of planting must be uniform to allow uniform plant growth. In hand planting, it is easier to plant at a spacing of 75 cm between rows and 60 cm between hills and 2 seeds per hill. Each plot has 42 plants of maize and 42 plants of green gram in alternate rows. The soil samples at surface level were collected from each plot and used for analysis prior to conducting the experiments after treating the soil with Cr and fertilizers.

### 2.2. Soil Analysis

Before the commencement of experiment, surface (0–20 cm) soil samples were collected at 3 sites from each plot using auger, pooled, air-dried, and 2 mm sieved for analysis. EC of the soil was examined by the conductivity meter. The measurement of pH, organic matter, and carbon of soil samples was done (1 : 25 soil and water ratio) by the ways of [29, 30]. The cation exchange capacity was analyzed by the technique of [31]. All chemicals were purchased from Merck (Germany) with appropriate purity analytical grade, and, during the experiments, the deionized water was used in the present study.

### 2.3. Seed Sterilization

The seeds of maize and green gram were obtained from seed centre in Palakarai, Kumbakonam, Tamil Nadu, India. Before starting experiments, seeds were soaked for 8 hours and placed in 10% sodium hypochlorite solution for 10 minutes for sterilization. Finally, they were rinsed with uncontaminated distilled water.

### 2.4. Experimental Design

The experimental design was completely randomized with 2 replications. Plots are arranged as follows:

 Control: water,
 Group I: water + FYM,
 Group II: water + NPK,
 Group III: water + FYM + NPK,
 Group IV: Cr,
 Group V: Cr + FYM,
 Group VI: Cr + NPK,
 Group VII: Cr + FYM + NPK.

The whole field is irrigated biweekly with tap water for maintaining water content of the soil to allow the heavy metal salt to reach a steady state.

### 2.5. Growth and Yield Data

At the harvest of 60 days (green gram) and 110 days (maize) after planting, six plants of each group were uprooted per plot to determine height, shoot, and root dry matter. The cobs were air dried and grain yield, weight of 100 kernels, and cob length were determined. For green gram, pod length, fresh and dry pod weight, moisture content (%) of seeds, seed yield/plant (g), and 100-seed weights were also analyzed. The number of nodules formed was determined further.

### 2.6. Nutrient Analysis of Seeds

In harvested green gram seeds, protein [32], carbohydrates [33], calcium by titrating with potassium permanganate [34], iron by dipyridyl method [34], and phosphorous [35] were estimated and, in maize, the seed carbohydrates and proteins were analyzed.

### 2.7. Metal Analysis

The harvested plant was taken with root and shoot, crushed into powder, and incinerated at high temperature. The ash obtained from the incinerated plant samples were treated with concentrated hydrochloric acid and the metals were analyzed using flame atomic absorption spectrophotometer (AAS) [36].

## 3. Results

### 3.1. Soil Analysis

Among all treatments, pH was paramount for FYM and lowest for NPK supply in both Cr treated and nontreated soil (Table 1). The pH of the soil is one of the significant properties for the mobility of heavy metals. When compared with controlled groups, total organic carbon (TOC) and organic matter (TOM) were found to be significantly increased in FYM treated soils. In particular, 70% increase in TOM was noticed after FYM application (Table 1) and the organic C level of the soil is enhanced by the presentation of organic matter in the form of FYM which has both direct and indirect effects on soil properties.

### 3.2. Growth Parameters

**Figure 1:**
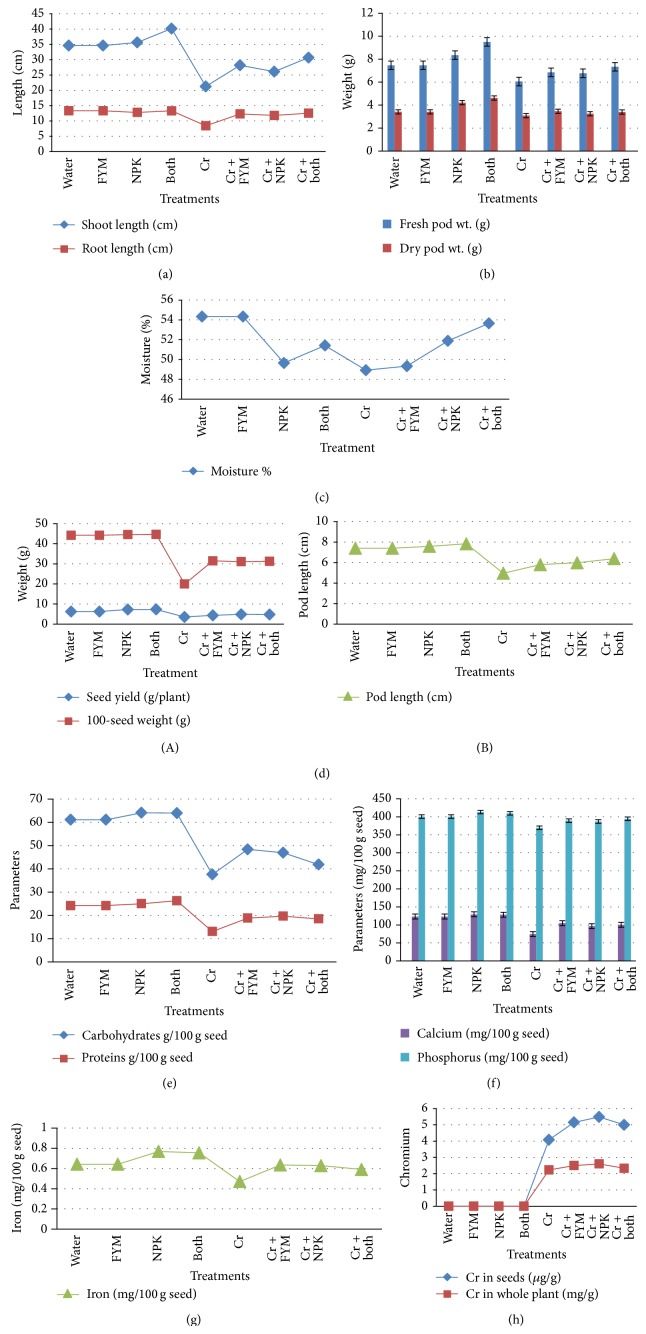
(a) Growth parameters of* Vigna radiata* under various experimental conditions. (b) Fresh and dry pod weight of* Vigna radiata* under various experimental conditions. (c) Moisture content of* Vigna radiata* under various experimental conditions. (d)(A) Yield parameters of* Vigna radiata* under various experimental conditions. (d)(B) Pod length of* Vigna radiata* under various experimental conditions. (e) Protein and carbohydrate of* Vigna radiata* under various experimental conditions. (f) Calcium and phosphorus content of* Vigna radiata* under various experimental conditions. (g) Iron content of* Vigna radiata* under various experimental conditions. (h) Metal accumulation capacity of* Vigna radiata* under various experimental conditions.

The shoot and root length of green gram plants after harvesting are given in Figure 1(a). The higher shoot (40.16 cm) and root length were recorded in the plants that received both FYM and NPK. There was a drastic reduction in shoot (38%) and root (37%) length of green gram which received Cr alone when it was compared with control. The application of fertilizer amendments increases the growth rate of the plants treated with and without Cr. The order of increase in such growth rate was Cr + NPK, Cr + FYM, and Cr + NPK + FYM.

**Figure 2:**
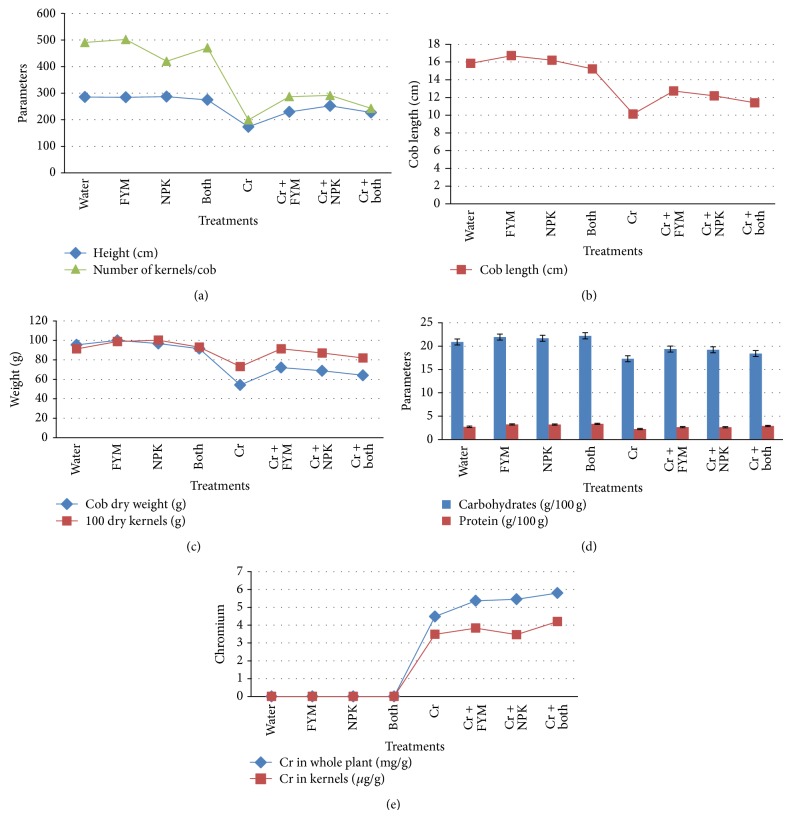
(a) Plant height and number of kernels of* Zea mays* grown under various experimental conditions. (b) Cob length of harvested* Zea mays* grown under various experimental conditions. (c) Yield parameters of* Zea mays* grown under various experimental conditions. (d) Protein and carbohydrate of* Zea mays* under various experimental conditions. (e) Metal accumulation capacity of* Zea mays* under various experimental conditions.

Total height of the maize plants of each group was measured to compare the growth rate under various amendments (Figures 1(a) and 2(a)). Among all amendments, NPK increased the growth rate in both Cr implemented (252.4 cm) and unimplemented (286.75 cm) plants. The results showed that Cr adversely affects the growth (173.3 cm) and the fertilizer amendments alleviate the toxicity caused by Cr and significantly increase the growth rate of plants.

### 3.3. Yield Parameters

The supply of Cr adversely affects the yield of green gram more than maize which indicates that it is more sensitive. The mean seed yield of green gram plants treated with Cr alone was found to be significantly (*P* ≤ 0.05) less (3.5 g/plant) than that of control (6.216 g/plant) group. The application of fertilizer amendments significantly increased the yield of green gram plants in the following order NPK > FYM + NPK > NPK (Figures 1(d)(A) and 1(d)(B)). Further, 100-seed weight green gram after harvest was determined to study the effect of Cr and fertilizer amendments on the yield. The maximum healthy yield was noticed in FYM (44.75 g) alone treated group and minimum was noticed in Cr alone treated plants (19.9 g). There was nearly 54% reduction in seed weight of Cr treated plants. The seeds with Cr treatment were unhealthy and very small in size. Fertilizer supply to the Cr treated seeds increases 100-seed weight. Cr + FYM treated seeds had 31.6 g, Cr + NPK showed 31 g, and in Cr + NPK + FYM 100-seed weight was noticed as 31.5 g. The pod length of different groups was also determined and it also varies significantly. In particular, the pod length of Cr treated group (4.9 cm) showed lesser length than that of all other groups (Figure 1(b)) and the moisture (%) is shown in Figure 1(c). The mean pod length of control plants was recorded as 7.4 cm. The application of fertilizers increases the size and weight of the pods in both Cr treated and untreated plants. While comparing Cr + FYM, Cr + NPK, and Cr + both with Cr treated plants, the yield and pod length were improved in the plants that received single fertilizer either NPK or FYM. Hence the application of both fertilizers is not necessary.

While analyzing the yield parameters of corn, the mean cob length of Cr treated plants was 10.13 cm which is lesser than that of control 15.85 cm. Fertilizer supply in Cr treated plants significantly increases cob length (Figure 2(b)). It is observed that the best result was found in FYM treated plants. The length of a cob is not a matter but number of kernels matters, so that was counted and compared. The total number of kernels in cobs of FYM treated plants was maximum (490) and Cr treated plants was minimum (199) (Figure 2(a)). The application of NPK in treated plants significantly increases the number of kernels (291). It may be due to better growth, development, and dry matter accumulation with proper supply of nutrient to plant and it increases the availability to other plant nutrients with the respective source of nitrogen application. The results indicated that Cr affects the growth and yield of corn even though it was reported as hyperaccumulator and tolerant for heavy metals (Figure 2(c)).

The size and nature of kernels were visually good and healthy even after Cr treatment. So the weight of 100 kernels of Cr + FYM treated and cobs were almost the same (91.2 g). Visually the seeds of green gram plants were unhealthy because of Cr treatment but corn kernels were not and the same as that of control. Finally with the results of yield it can be suggested that the Cr treatment affects the yield of plants but amendment of FYM or NPK with corn in mixed crop cultivation improves the yield.

### 3.4. Nutrient Content

Protein, carbohydrate, calcium, phosphorus, and iron of harvested green gram seeds and protein and carbohydrate of corn kernels were analyzed (Figures 1(e), 1(f), 1(g), and 2(d)). The carbohydrate content background values for green gram and maize plant are 63 g/100 g and 31 g/100 g, respectively. In maize, mean corn carbohydrate content of control plants was recorded as 20.9 g/100 g and protein as 2.73 g/100 g. Cr treatment slightly reduces the carbohydrate and protein to 17.3 g/100 g and 2.2 g/100 g, respectively, in the same group. The protein content background values for green gram and maize plant are 24 g/100 g and 4.3 g/100 g, respectively. The average protein content of maize was found to be approximately 3.2 g/100 g in control, FYM, NPK, and FYM + NPK treatments. Carbohydrate content of the same groups vary from 20 to 22 g/100 g. NPK + FYM showed best result for protein (2.9 g/100 g) content among the groups that received Cr and fertilizers. For carbohydrates FYM gave good results (19.3 g/100 g) and the results are approximately equal to the control plants. Carbohydrate and protein content of green gram seeds in control group were found to be 61.08 mg/g and 24.25 mg/g, respectively. Cr application severely affects the nutrient content of green gram seeds (carbohydrate 37.5 mg/g and protein 13.11 mg/g) but it was not much affected in maize (Figure 2(d)). In green gram seeds Cr treated plants which received FYM had high carbohydrate (48.4 mg/g) among three fertilizer amendments and protein was high in NPK received Cr treated plants (19.68 mg/g). This may be due to the high availability of nitrogen for protein synthesis.

Fe and Ca level of Cr treated green gram seeds showed approximately 50% reduction when they were compared to control. Treatment of plants with fertilizers helped the plants to retain the nutrient level. Better results were found in Cr + FYM treated plants for Fe (0.635 mg/100 g) and Ca (105 mg/g). The phosphorous content was increased in NPK treated green gram plants and this may be due to the availability of P in more amounts. There is no significant difference in the rest of the treatments.

### 3.5. Metal Content

The amounts of Cr in whole plant and in harvested seeds of both plants were presented in Figures 1(h) and 2(e). Metal uptake was enhanced by NPK + FYM in maize (plant: 5.8 mg/g, seeds: 4.2 g/g) and in green gram NPK treated plants showed high metal content in both whole plant 5 mg/g and in seeds (2.3 g/g). Cr alone treated green gram plants accumulated 2.2 mg/g and seeds 5 g/g. Comparing this with the corn plants, there is a substantial difference in the Cr level of both plants (4.48 mg/g) and seeds (3.48 g/g). Green gram plants are accumulating higher Cr in seeds than that of maize seeds.

## 4. Discussion

### 4.1. Soil Analysis

FYM increases the organic content of soil and subsequently the pH of soil owing to the discharge of humic substance is increased and humic acid is formed through decomposition. pH in river sand amended with FYM was increased [37]. Further, NPK application showed the lowest pH. The phosphate in NPK increases the development of dicalcium phosphate (slowly soluble) with a release of phosphoric acid, declining the soil pH through its conversion to phosphorus and hydrogen ion. The super phosphate fertilizers decrease the soil pH [38]. FYM was found to be a normal source of organic content [39].

### 4.2. Growth Parameters

When metal content of soil becomes high, the plant loses its role, possibly because of the lethal exploit by the metal, and the uptake especially increases. Further, because of improved uptake, metals interrelate with various cellular mechanisms and disturb the normal metabolic reactions, producing cellular damage and the death of the plants in severe cases. The higher concentrations of copper and cadmium had the most phytotoxic effects on green gram plants, probably owing to the inhibition or breakdown of biomolecules including enzymes, proteins, and DNA, through the generation of reactive oxygen intermediates [40].

The eventual caustic effect is mainly because of singlet oxygen and hydroxyl radicals. All classes of biomolecules are damaged by the specific and quick reaction of these radicals. The oxidative strain in pea leaves because of cadmium treatment has been stated [41]; in bean, copper is known to interact with oxidative enzymes [42]. Further, the decrease in plant growth could be owing to reduction of photosynthetic pigments [43] and Rubisco activity [44]. The chromium toxicity in plants occurs by inhibiting the growth more or less, showing chlorosis with brownish red or purple leaves and necrotic lesions. High chromium concentration inhibits photosynthesis and seriously inhibits the root growth [45].

### 4.3. Yield Parameters

Related results have been stated for the yield of maize developed in sewage sludge and MSW compost [46] when it is compared with fertilizer-amended soil. Further, MSW composts loaded in a wide ranging of plant nutrients can considerably improve the plant development [47]. Bisessar [48] reported that the decrease in shoots and roots was suppressing both dry matter production and seed yield. The drop in seed yield after applying heavy metals has been accredited to the toxic effects of metals on the propagation of roots and shoots [49]. The characters of the plant vary under various treatments. FYM increases the organic carbon of the soil and improves both soil quality and growth of plants [50]. Wilden et al. and Cogliastro et al. [51, 52] have witnessed that high organic matter and macro- and micronutrients enhanced both the soil properties and the plant yield. Yadav et al. and Abrol et al. [53, 54] have reported enhanced accessibility of all type of nutrients in soil because of organic and inorganic combinations.

### 4.4. Nutrient Content

Bhattacharyya et al. [55] reported that FYM amendment showed better supply of nitrogen, phosphorus, potassium, and enhanced soil physical environment than unamendment ones. The reduction in protein content may be due to the poor availability of nitrogen [56]. The reduction in the nutrient content may be due to the inhibition of enzymes involved in the synthetic process [57].

Ranganathan and Salvseelan [58] stated that FYM improved the availability of N, Fe, and Zn and P in soil. Adding of organic materials increases intractable fraction of phosphorus. Microbial activity also increased and thus biochemical transformation [59]. The connections with the soil mechanism to increase phosphorus uptake by the plants are most important [60].

### 4.5. Metal Content

The application of fertilizers slightly increases the metal accumulation in both the plants and alleviates the toxicity of metal by providing required nutrients to the plants. This result is in accord with previous studies [61]. Fertilizer effects on metal uptake of plants may result from a change of metal availability in soil. Root exudates containing organic acids form complexes with Cr, making them available for plant uptake. Soil treated with FYM and NPK indicated higher positively charged ions, due to disintegration products of FYM in soil that have detained the fixation of the useful fertilizer [62].

## 5. Conclusion

Thus, the results of the present study concludes that, amongst all treatments, availability of chromium was higher only in NPK and FYM modified soil for maize and NPK soil modified for green gram. Plants grown in FYM and NPK alone showed better growth than that of test control. The present study proposes that FYM (organic) or NPK (inorganic fertilizer) may be applied to lessen the toxicity of chromium in the soil and to maintain the strength of physiological growth of plants. FYM or NPK application also improves the growth and yield of plants in polluted sites used to horticulture and agriculture processes in mixed crop cultivation.

Proteomic analysis of harvested seeds of maize and green gram is is not done in the present study and it is under process. Further, Cr recovery from plants and seeds will be concentrated in the future.

## Figures and Tables

**Table 1:** Physicochemical properties of soil collected from the experimental field before planting.

Properties	Water	FYM	NPK	FYM + NPK	Cr	Cr + FYM	Cr + NPK	Cr + FYM + NPK
pH	7.2	7.7	6.9	7.3	6.7	7.4	6.9	7.0
EC (dS/m)	0.14	0.13	0.15	0.15	0.33	0.26	0.23	0.21
Organic matter (%)	1.6	2.3	1.9	2.3	1.6	2.3	2.0	2.2
Organic carbon (%)	2.5	2.8	2.3	2.7	2.4	2.9	2.3	2.4
CEC (mmol/100 g)	9.5	9.3	9.1	9.3	8.6	9.2	8.9	9.2
